# Foreign Body in the Airway Mimicking Tumour in an Adult: A Case Report

**DOI:** 10.7759/cureus.58584

**Published:** 2024-04-19

**Authors:** Róbert Šimon, Jana Šimonová, Lukáš Čuchrač, Roman Klimčík, Janka Vašková

**Affiliations:** 1 1st Department of Surgery, Pavol Jozef Šafarik University, Košice, SVK; 2 1st Department of Anaesthesiology and Intensive Medicine, Pavol Jozef Šafarik University, Košice, SVK; 3 Department of Pneumology and Phthiseology, Pavol Jozef Šafarik University, Košice, SVK; 4 Department of Medical and Clinical Biochemistry, Pavol Jozef Šafarik University, Košice, SVK

**Keywords:** high-frequency ventilation, intensive care, empyema of the chest, bronchoscopy, foreign body aspiration

## Abstract

Foreign body (FB) aspiration is an infrequent cause of respiratory distress in adults. Advancing age, central nervous system disorders or trauma, drug or alcohol addiction, neuromuscular diseases, and mental health issues and illnesses are the main risk factors. The authors present an atypical clinical presentation of a 3-week-lasting foreign body aspiration mimicking a tumour that led to severe acute respiratory insufficiency and required aggressive artificial lung ventilation. Diagnosis of FB was based on the results of the chest computed tomography (CT) scans and flexible bronchoscopy, which, however, initially assumed a neoplastic disease in the right main bronchus. During FB extraction via flexible fiberoptic bronchoscopy inserted through an 8.5 mm endotracheal tube high-frequency ventilation through a catheter placed between the vocal cords was used to ensure adequate alveolar ventilation and maintain sufficient oxygenation. After extraction of the FB, thoracosurgical intervention was performed to resolve empyema as a septic complication of the FB aspiration. After this therapy, a complete resolution of pleural empyema and lung atelectasis was observed.

## Introduction

Foreign body (FB) aspiration into the airway is not frequent in the adult population. Adults represent up to 25% of all cases [1]. Patients over the age of 65 years old are less likely to remember the aspiration [1]. Organics, especially bone fragments, and seeds are the most aspirated FBs by adults [2]. Typical symptoms consist of choking, coughing, dyspnoea, and hemoptysis. However, these findings are non-specific and may mimic chronic lung diseases or tumours [1]. Therefore aspiration of an FB in adults is frequently misdiagnosed [3]. Patients may also present with acute respiratory failure requiring urgent intervention [4]. The majority of FBs become lodged in the right bronchial tree, favoring the bronchus intermedius and the basal segments of the right lower lobe [1]. The chest X-ray and computed tomography (CT) can provide information regarding the location and characteristics of the foreign bodies, but the golden standard for diagnosis and management of FB extraction is bronchoscopy [1]. Flexible fiberoptic bronchoscopy is a rapid, cost-effective, and safe procedure and often is chosen as the first procedure to remove the airway FB. Rigid bronchoscopy is used as a second-line procedure [3]. Delay in diagnosis can lead to serious complications such are pneumonias, lung abscesses, empyema, or other complications and thus increase morbidity and mortality rates. 

## Case presentation

We present a case report of a 57-year-old man, a smoker, with intellectual disability and obesity (his BMI value was 121 kg/1.75cm^2^ = 39.54). He had a history of chronic obstructive pulmonary disease. The patient was initially admitted to the Emergency Department on 4 April 2019 for several days of progressive dyspnoea. The patient inhaled oxygen 2 liters per minute through the face mask, pulse oximeter values ranged from 91-92%. Hypercapnia without severe respiratory acidosis and hypoxemia was seen in the capillary bedside acid-base measurement (pH 7.34, partial pressure of carbon dioxide (pCO_2_) 8.9kPa, partial pressure of oxygen (pO_2_) 9.7kPa, bicarbonate (HCO_3_) 29.2 mmol/l, and standard base excess (SBE) 5.6 mmol/l). ​​​The patient remained conscious. There was severe dyspnoea, tachypnoea without cyanosis, and coughing during initial exertion. ​​Turgor of the skin was normal, breathing was vesicular with prolonged expiration. He was haemodynamically stable. Laboratory investigations showed increased levels of inflammatory markers (Table 1). Subsequently, the oxygen flow was increased to 3-4 liters per minute, and pulse oximeter values increased up to 97%, but his hypercapnia worsened, and for that, oxygen flow was decreased to 2 liters per minute. 

**Table 1:** The blood inflammatory parameters on 4 April 2019 CRP - C-reactive protein, PCT - Procalcitonin, WBC - white blood cells, ANC - absolute neutrophil count

Parameter	Levels
S-CRP	141.54 mg/l
S-PCT	0.01 µg/l
WBC	10.8x10^9^/l
ANC	8.34x10^9^/l

No infiltration was found on chest X-ray. The patient was admitted to the ICU of the 1st Department of Internal Medicine. Empiric antibiotic treatment was started with cefotaxime 6g/day. The values of acid-base parameters were worsened. Non-invasive ventilation (NIV) through a full face mask was started. The initial settings were as follows: PS (pressure support) 5-10 cmH_2_O, PEEP (positive end-expiratory pressure) 8 cmH_2_O, and fraction of inspired oxygen (FiO_2_) 0.35-0.40. Due to the progression of hypercapnia and hypoxemia (Table 2) and deterioration of consciousness despite NIV, the patient was transferred to the 1st Department of Anaesthesiology and Intensive Medicine on 5 April 2019.

**Table 2:** Trend of acid-base parameters during the first 48 hours after admission NIV - non-invasive ventilation, OTI - orotracheal intubation, FiO_2_ - fraction of inspired oxygen, pO2 - partial pressure of oxygen, pCO2 - partial pressure of carbon dioxide, SBE - standard base excess, stHCO3 - standard bicarbonate

Date	Time	Mode of O_2_ delivery	pH	pO_2_ (kPa)	pCO_2_ (kPa)	SBE (mmol/l)	stHCO_3_^-^ (mmol/)l	sat (%)
04/04	7:00	Face mask 2 liters per minute	7.340	9.7	8.9	5.6	29.2	92%
04/04	7:40	Face mask 4 liters per minute	7.270	15.6	10.00	6.8	28.1	97.4
04/04	19:00	NIV (full face mask)	7.382	5.34	8.02	9.6	31.3	74.8
05/04	7:34	Before OTI	7.172	5.59	14.5	9.9	38.4	67.8
06/04	7:20	After OTI (FiO_2_ 0.8)	7.359	7.51	9.05	11	32.8	89.1
06/04	15:40	OTI	7.492	11.4	5.11	5.6	29.6	97.8

After admission, he was immediately intubated and connected to artificial lung ventilation with aggressive initial parameters (FiO_2_ 0.80, pressure control (PC 26 cmH_2_O), PEEP 10 cmH_2_O). Moxifloxacin was added to cefotaxime. Diminished or absent breath sounds on the right side were present during lung auscultation. Ultrasound examination of the chest showed a 3 cm pleural effusion. Flexible bronchoscopy was performed and an exophytic tumour was found in the right main bronchus 1 cm below the tracheal carina with nearly total obstruction. 

**Figure 1:**
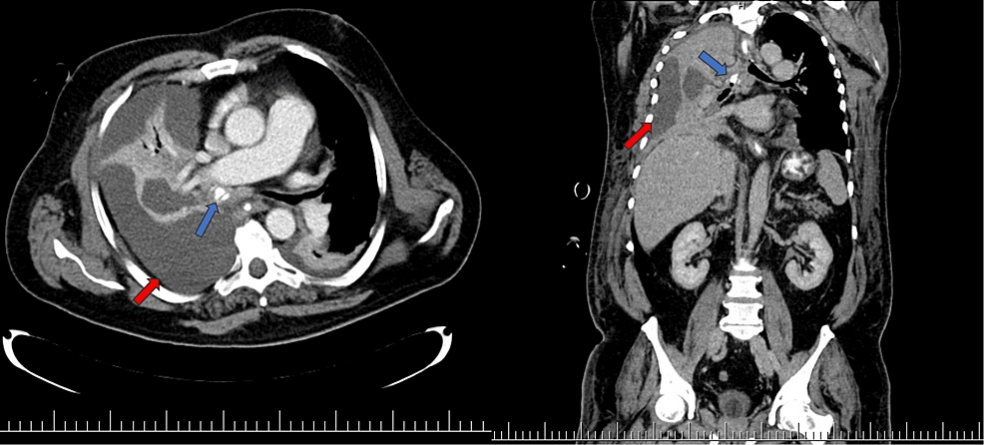
Axial and coronal CT image of the lung with intravenous contrast on 8 April 2019 revealing a calcifying lesion in right main bronchus (blue arrows) and pleural effusion (red arrows)

Lung and mediastinum computed tomography for suspicious malignancy was performed. An intraluminal obturating 27x11 mm tumour of the right main bronchus was diagnosed with complete atelectasis of right lung with hilar and mediastinal lymphadenopathy. In addition, right pleural effusion was seen (Figure 1). 

**Figure 2:**
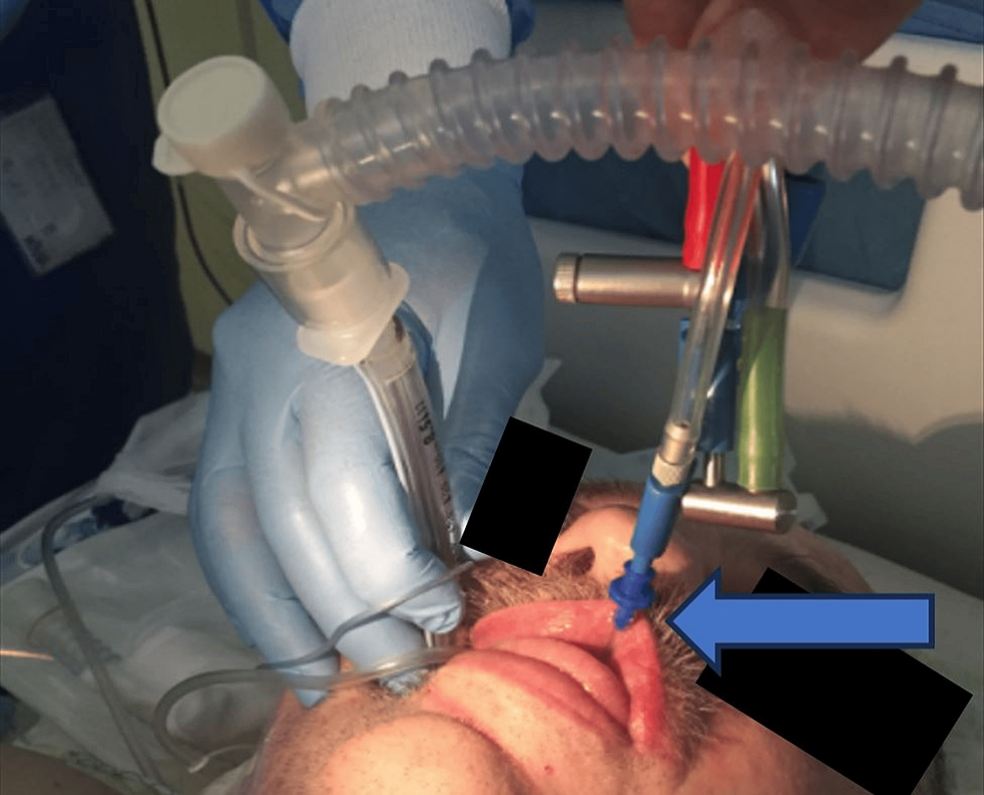
High-frequency ventilation (blue arrow) during bronchoscopic extraction of the foreign body

The next day, flexible bronchoscopy was performed by a pneumologist to take a biopsy from the tumour, and FB aspiration was suspected for the first time. But its extraction failed. On the same day, a rigid bronchoscopy was performed. Extraction of the FB under general anaesthesia along with muscle relaxation was unsuccessful due to severe hypoxemia during the procedure despite the fact that we pre-oxygenated the patient with 100% oxygen. The following day, flexible bronchoscopy was performed under general anaesthesia with high-frequency jet ventilation through a catheter. Under direct laryngoscopy, the 14 gauge catheter was orotracheally inserted 5 cm below the vocal cords and connected to a special ventilator to prevent hypoxemia during the procedure (Figure 2).

**Figure 3:**
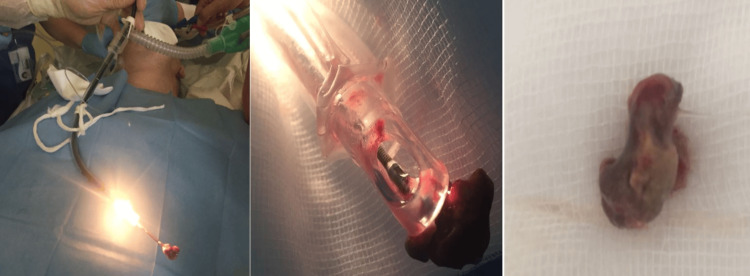
The endobronchial FB (chicken bone) extracted via flexible fiberoptic bronchoscopy together with endotracheal tube (diameter of the FB was larger than the diameter of endotracheal tube). FB - foreign body

Granulation tissue was seen together with a bone fragment. A part of a chicken bone was extracted with a rat-toothed extractor together with an endotracheal tube because of the larger diameter of the extracted FB (Figure 3). Debridement of granulation tissue and bronchus recanalization, together with drainage of the right pleural effusion (empyema) were also performed. The patient was reintubated and connected to artificial lung ventilation. The high-frequency jet ventilation catheter was removed. 

**Figure 4:**
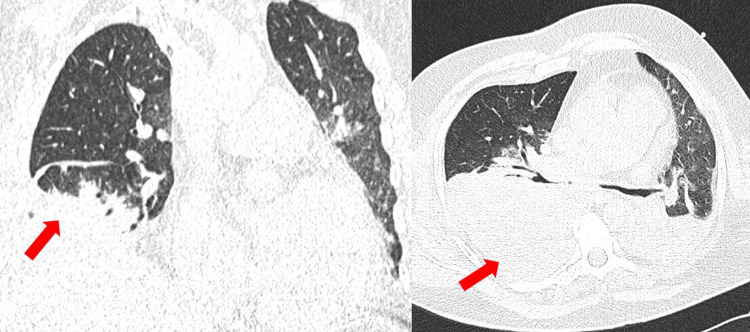
Coronal and axial CT image of the lungs without intravenous contrast on 10 April 2019 after extraction of the foreign body revealing persistent atelectasis and pleural effusion (red arrows).

The course was complicated by persistent atelectasis of lung segments S6, 2, 9, and 10 with persistent pleural effusion- empyema (Figure 4). 

After preoperative preparation, right muscle-sparing thoracotomy was performed in the 5th intercostal space, with pleural effusion evacuation, lung decortication, and pleural drainage. General anaesthesia with one-lung ventilation was used. A double-lumen tube was inserted into the left main bronchus. 

**Figure 5:**
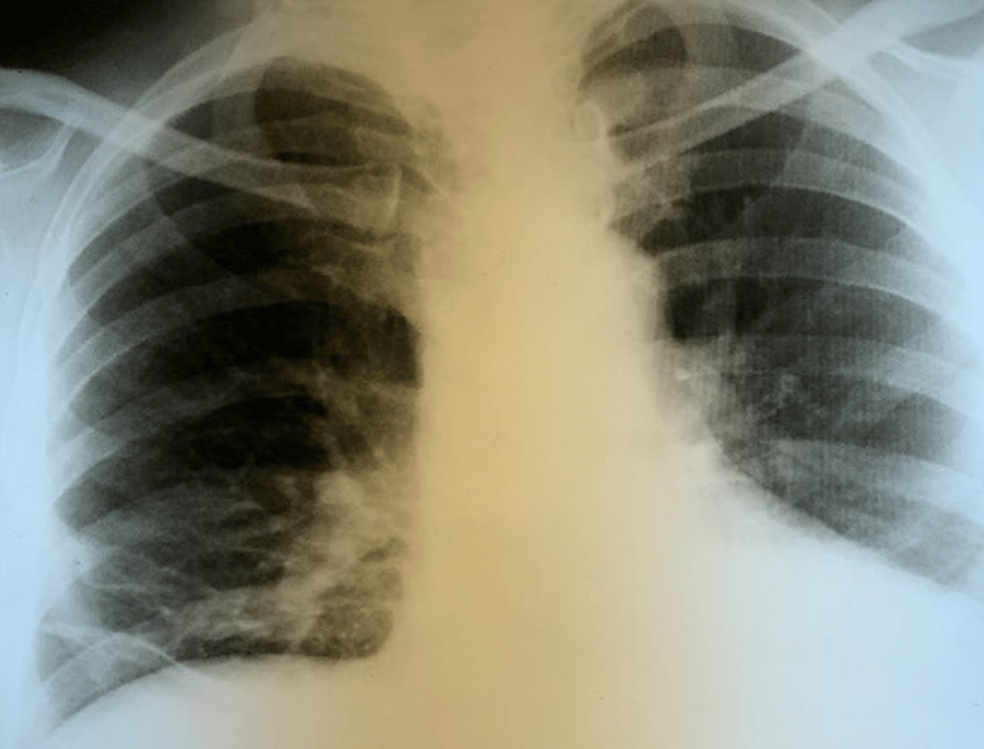
Chest X-ray on 14 August 2019

After thoracotomy, artificial lung ventilation and antibiotic treatment continued. On 18 April 2019 percutaneous dilatation tracheostomy was performed. Postoperative care was without complications, and antibiotic treatment was changed according to the results of microbiological examinations. Inflammatory parameters decreased, chest X-ray was without pneumothorax and pleural effusions. On 8 May 2019 the patient was weaned from artificial lung ventilation, he was decannulated on 13 May 2019 and discharged from the hospital on 30 May 2019. The patient had been following up with the Department of Pneumology and Phthiseology without further complications. There were only postoperative adhesion changes on chest X-ray (Figure 5) and the tracheostomy wound showed healing. At the time of manuscript preparation, the patient is self-sufficient and continues to be checked by a pneumologist on a regular basis.

## Discussion

Foreign body aspiration (FBA) is relatively infrequent in adults. Most publications on this topic are case reports, and there are very few case series. The most common types of airway FBs (AFBs) were food material (66%) [4]. In adults, organic FBs, mainly bone fragments (especially chicken), fishbones, pieces of vegetables or fruits (cherry pits or plum stones), and seeds (melon or sunflower) are more frequent. There is a much higher proportion of iatrogenic foreign bodies (parts of dentures or tracheal cannulas) in geriatric patients [5]. Relevant history-taking is a very important step in the diagnosis of FBA. However, the most frequent risk factors of FB aspiration in adults are older age, abuse of alcohol or sedative medications, neurological disorders, intellectual disability, trauma with loss of consciousness, and tracheostomy cannula handling [3,6,7]. In these cases, relevant history-taking can be difficult. A history of aspiration is not found in the majority of adult patients. In these cases and when patients cannot verbally explain symptoms, the diagnosis of FB aspiration cannot be made. The diagnosis can be undetected even for years [8]. Patients over the age of 65 years are less likely to remember the aspiration event, with only 30% supplying a history consistent with an aspiration event prior to bronchoscopy [9].

Aspiration may present in different ways [10], and it can be life-threatening. Symptoms are determined by the size of the FB and the location in which it becomes lodged. Impaction in the trachea leads to a more dramatic presentation of inspiratory stridor with frequent coughing, while occlusion of the lower bronchi can result in coughing, wheezing, dyspnea, or hemoptysis, and may be mistaken for an alternative diagnosis [11]. Although very common, aspiration is rarely considered in the absence of an acute clinical presentation. In adults, the classical symptoms of coughing, dyspnoea, choking, wheezing, and cyanosis occur in only a small percentage of patients. The most frequent clinical symptoms are cough, purulent sputum production, hemoptysis, and fever [3,12], all nonspecific clinical signs. This is called a non-asphyxiating FB aspiration [6]. These nonspecific symptoms can mimic other respiratory diseases like asthma, bronchitis, pneumonia, or lung tumour [5]. Also, tuberculosis, epiglottitis, retropharyngeal abscess, peritonsillar abscess, postviral pericarditis or pleuritis, and bronchiolitis may present with clinical findings similar to foreign body aspiration. Traumatic injuries with localized pulmonary, airway, or even diaphragmatic injury may present similarly to foreign body aspiration [1,10]. The lack of precise history and paucity of symptoms often results in misdiagnosis or delayed diagnosis [9,13]. 

Ramos et al. analysed 12 publications focused on FB aspiration in adult patients. They found that FB aspiration in adults is more frequent in the right bronchial tree (71,5% vs 22,8%) [2]. Lodgement was more common in the bronchus intermedius (27%) and right lower lobe (33%) [2,3]. The same results were published by Ng et al. - 61% of AFBs were in the right bronchi [4]. This could be explained by a more direct pathway to the right main stem bronchus [3]. However, due to the more direct course of the right bronchus, the probability of coughing up FB may also be higher.

Imaging techniques are of importance in establishing the diagnosis of FB aspiration [5]. Posteroanterior chest X-ray is the standard diagnostic method. Metallic FBs are easily identified but are less frequent [2]. Obstructive emphysema, alveolar infiltrates, atelectasis, and mediastinal shift are suggestive of FB aspiration [11]. Sancho-Chust et al. demonstrated that the most frequent radiological findings were alveolar infiltrates and atelectasis [12]. Chest CT can help in detecting the foreign body. The most reliable is a demonstration of the FB in the tracheobronchial tree. Nonspecific findings are thickened bronchial wall next to FB, pneumonic infiltrates, bronchiectasis, atelectasis or lobar consolidation, ipsilateral pleural effusion, and hilar lymphadenopathy. These findings on CT have to indicate a bronchoscopy. Flexible bronchoscopy is effective in the diagnosis and removal of FBs [14]. Ng et al. analysed 103 patients with AFBs. Chest radiographs were normal for 26 (25%) patients. The majority of patients (81%) had a visible aspirated FB on chest CT scans [4]. 

AFBs can be removed by flexible bronchoscopy or rigid bronchoscopy [4]. There are few reports comparing flexible and rigid bronchoscopy in adult FB aspiration [3,4]. Flexible bronchoscopy is a rapid, cost-effective, and safe procedure [3]. Rigid bronchoscopy can be a backup procedure in cases where flexible bronchoscopy has failed [10]. In some rare cases, thoracotomy was performed for patients whose FB removal was unsuccessful even with rigid bronchoscopy [3]. Flexible bronchoscopy is generally regarded as a safe procedure. In critically ill patients with severe hypoxemic respiratory failure requiring mechanical ventilation, it could be a challenge because critical hypoxemia may occur. High-frequency ventilation through a catheter inserted below the vocal cords could be useful to maintain adequate ventilation and oxygenation during the procedure. 

Most of the patients had no complications. However, a myriad of complications, including recurrent pneumonia, bronchiectasis, lung abscess, and atelectasis, can occur from a missed FB aspiration [15]. Complications of FB aspiration are associated with the type of FB and the interval between aspiration and removal. Vegetable or organic materials could cause a higher rate of complications in that proteins or lipids could lead to more severe inflammation and tissue injury. The longer the FB stayed inside the airway, the higher the complication rate was [3]. Ng et al. found that the median duration between aspiration and bronchoscopic removal of the aspirated FB in their hospital was 21 days (3-129 days) [4]. A neglected aspirated FB can lead to complications that are sometimes difficult to manage, dramatically increasing the morbidity and mortality rate. In our patient, after further questioning, his mother reported that he had eaten chicken soup greedily with mild choking about 3 weeks before being admitted to the hospital. Undiagnosed and retained aspirated FBs may result in serious delayed complications such as recurrent pneumonia, obstructive emphysema, bronchial stenosis, bronchiectasis, pneumothorax, pneumomediastinum, recurrent haemoptysis, pleural effusion, empyema or bronchopleural fistula and thoracosurgical interventions are often required [3].

## Conclusions

We present a case report of unrecognized FB aspiration in a patient with intellectual disability, so the history of aspiration was difficult to obtain. The clinical course deteriorated rapidly, and intubation and artificial lung ventilation were needed. The diagnosis was based on a CT scan of the lung and flexible bronchoscopy. High-frequency ventilation through a small 14-gauge catheter inserted below the vocal cords was used to maintain sufficient ventilation and oxygenation of the patient during fiberoptic bronchoscopy and extraction of the foreign body. 
